# Arteriovenous Malformation of the Lip: A Rare Case Report

**DOI:** 10.7759/cureus.8979

**Published:** 2020-07-03

**Authors:** Shaul Hameed Kolarkodi, Ayoob M Alnafisah

**Affiliations:** 1 Maxillofacial Surgery and Diagnostic Science, Qassim University, Qassim, SAU; 2 Dentistry, Qassim University, Qassim, SAU

**Keywords:** arterio-venous malformation, color doppler‎, hemangioma‎, sclerotherapy‎

## Abstract

Vascular malformation (VM) consists of a group of tumors that emerge from vascular origin caused by vascular angiovascular or lymphoproliferation. Arteriovenous malformations (AVMs) contribute high-flow, creating direct vein artery contact without regular capillary network. AVMs are present at birth or in congenital. Acquired AVMs occur later in life due to hormonal changes or trauma, and acquired AVMs in oral cavity are very rare. AVMs are persistent and progressive in nature when present, can represent a lethal, causing significant blood loss, and an incomplete resection frequently leads to a recurrence of the lesion. We present a rare case of a congenital AVM diagnosed on retinue dental checkup using color doppler ultrasonography (USG) in a 62-year-old man from South India.

## Introduction

Arteriovenous malformations (AVMs) are the lesions with direct communications between endothelial‑lined artery/arteries and vein/veins bypassing the capillary bed [1]. Head and neck AVMs are reported to occur in 0.1% of the population of which extracranial accounts for only 8.1% [2]. AVMs may be congenital or acquired. Acquired AVMs are usually post-traumatic with previous history of injury, trauma, or surgery with higher incidence of hemorrhage. Hemorrhage and disfigurement are common reasons requiring intervention in AVMs involving the head and neck region [2].

Arteriovenous malformation is congenital in nature and consists of a fistulae-tangle of arteries and veins, where the central component is a nidus. Hemorrhage is the most common symptom but other symptoms include headache, seizures, stroke-like symptoms, and ischemic stroke. Within the literature, cognitive results are rarely published, with some indications of a progressive course among case series reports [3]. Hence, we report here a case of AVMs in a 62-year-old South Indian man diagnosed on a retinue dental checkup using ultrasonography (USG) with color Doppler application and discuss the need for timely care for this case.

## Case presentation

**Figure 1:**
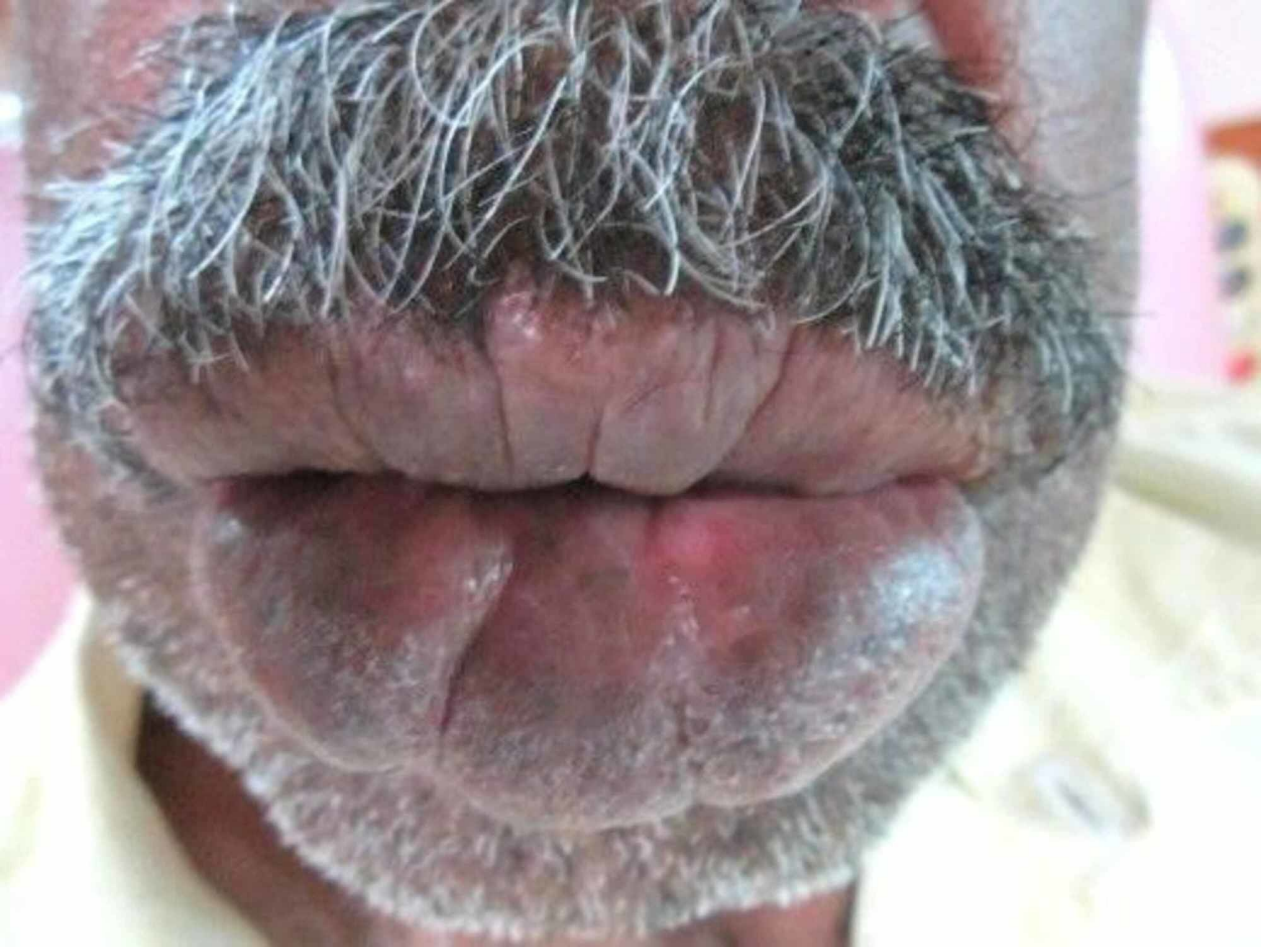
Swollen upper and lower lip‎.

A 62-year-old male patient presented to the Department of Oral Medicine and Radiology for the retinue dental checkup. Extra oral examination revealed swollen lips, history revealed that it started at birth and slowly progressed, asymptomatic, no history of bleeding or discharge (Figure 1).

**Figure 2:**
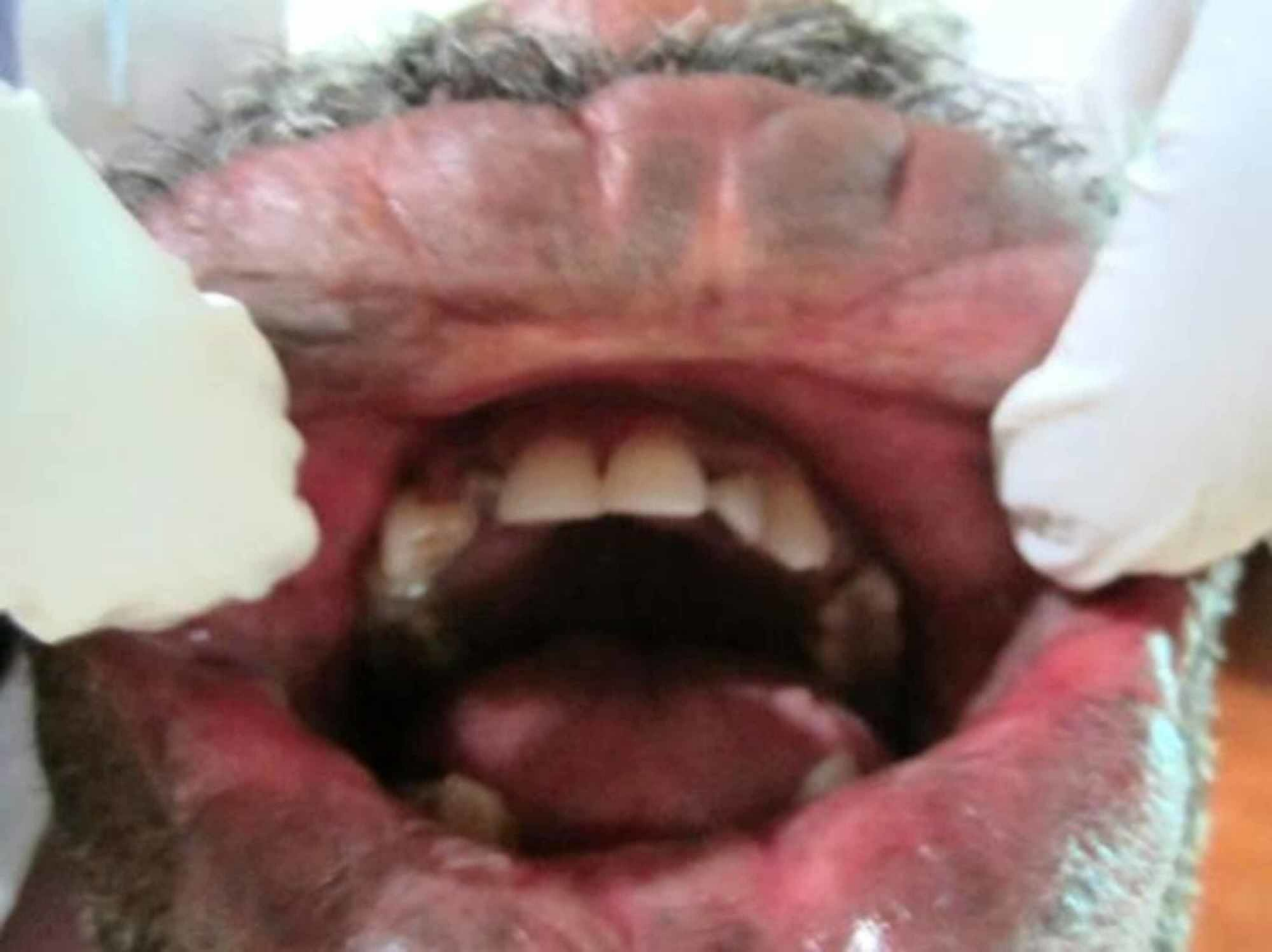
Entire upper lip with a lesion of 2.5 cm.

**Figure 3:**
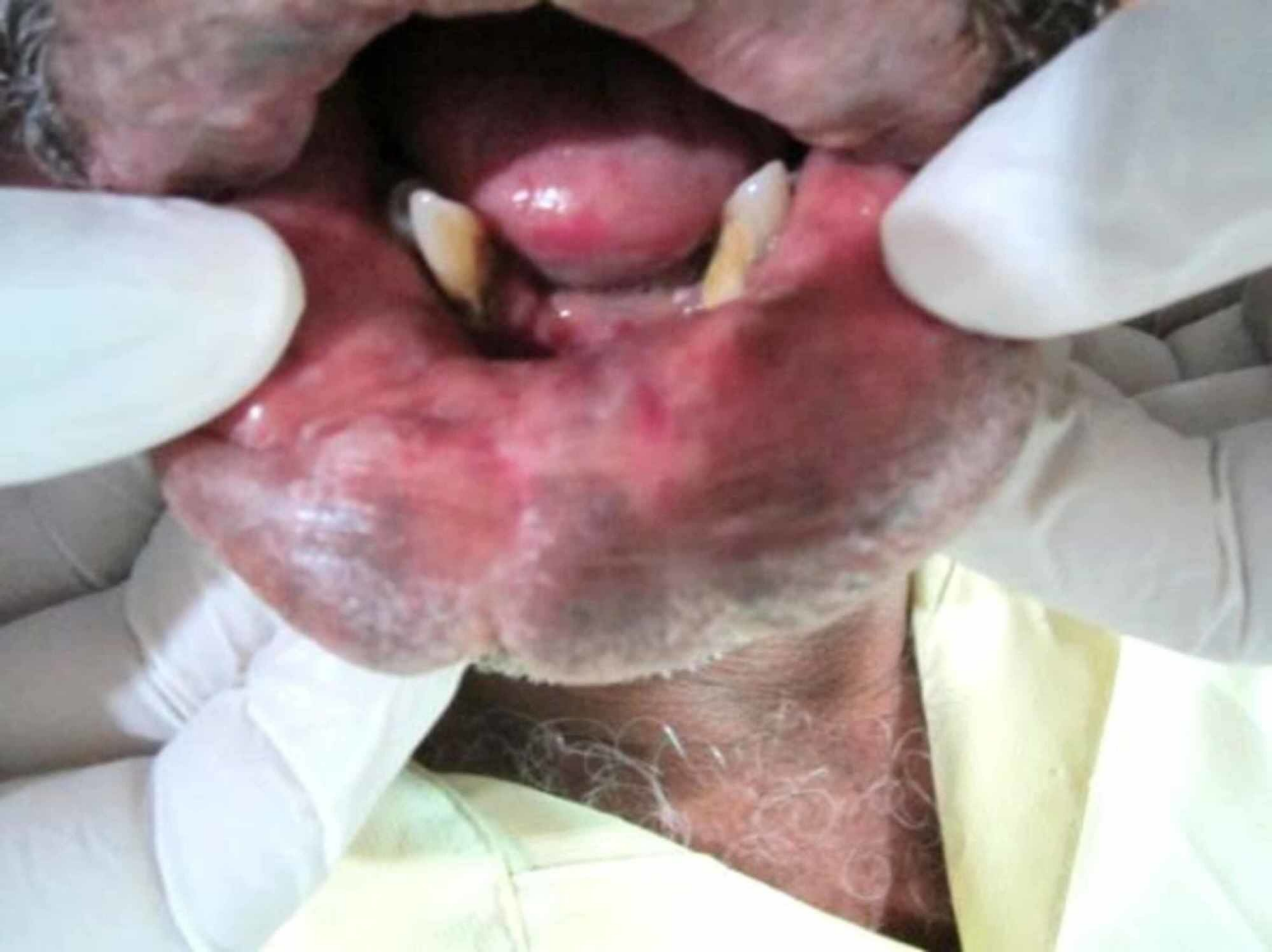
Entire lower lip is involved with a thickness of 2.5 cm‎.

The intraoral clinical examination revealed over all swelling of the both upper and lower lips, with normal color, measuring 2-3 cm thickness of the lips; on palpation it was painless, fluctuant, nonreducible, and compressible with the presence of pulsatility. Furthermore, on chair side diascopy test blanching was noted (Figures 2-3).

**Figure 4:**
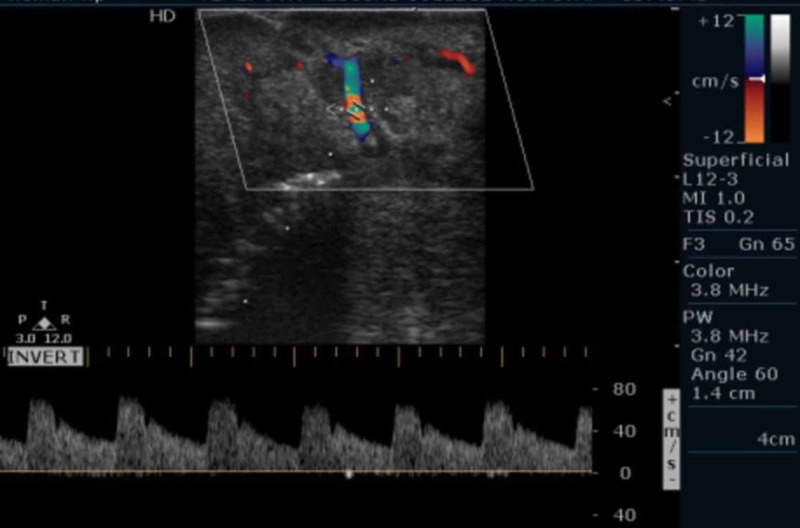
Color doppler application on USG showing turbulence of blood flow signals on the bottom suggesting AVM.‎ USG, ultrasonography; AVM, arterio‎venous malformation

**Figure 5:**
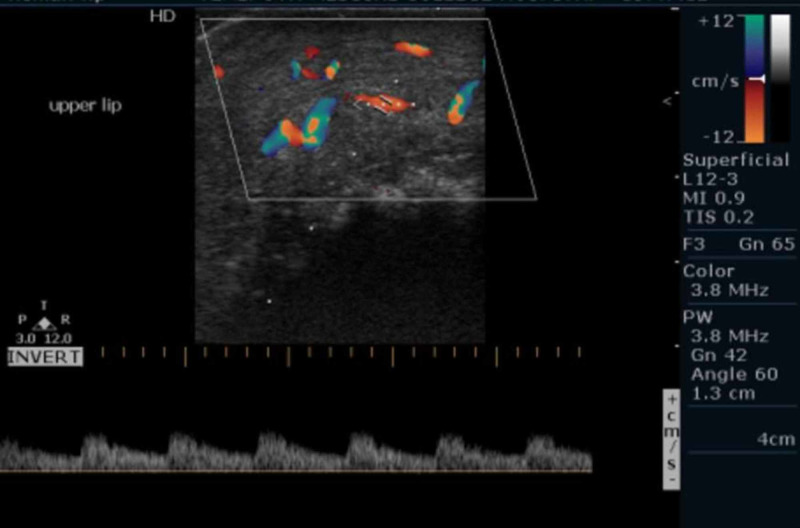
Color doppler application on USG showing moderate uptake by mixed blue and red color suggesting AVM.‎ USG, ultrasonography; AVM, arteriovenous ‎malformation

Based on the clinical examination and chair side investigation provisional diagnosis of hemangioma of the lips was made and differential diagnosis of congenital, acquired, and familial arteriovenous malformations was considered. But detailed history of onset at birth and generalized involvement of entire upper and lower lip favored a clinical diagnosis of congenital AVMs. The patient was subjected to USG with color Doppler application; it presented turbulence of blood flow vascular signals within the lesion and moderate amount of color uptake both red and blue suggestive of the AVMs means capillary as well as cavernous malformation (Figures 4-5).

The patient was informed about the diagnosis treatment options, the risk in carrying out biopsy, and the dental surgical procedure. Histopathology helps in final diagnosis but high risk of bleeding; its advised to carry it after identification and embolization of feeding vessel using contrast CT and later the patient was referred to a higher center for further investigation and management.

## Discussion

Vascular abnormalities are a heterogeneous group of vessel disorders that may affect any section of the vascular tree; arteries, capillaries, veins, or lymphatics, or a combination of these [1]. Each anomaly is characterized by its unique anatomy, pathophysiology, clinical behavior, and approach to management [1-2]. They are among the most complicated diagnostic and therapeutic enigmas in the area of the head and neck [3-4]. Hence, developing a classification system that will address both diagnostic and therapeutic problems is of utmost importance. Virchow and Wagner provided early classification according to the vessel's pathological appearance. They subclassified vascular growths into angiomas and lymphangiomas. The biological actions of the vascular lesions and their natural history were not considered [4]. In 1982, Mulliken and Glowacki introduced into two main categories a biological classification based on their clinical appearance, histopathologic characteristics, and biological behavior: tumors and malformations [1, 5]. This classification was later redefined by Mulliken and Young, and approved in 1996 by the International Society for the Study of Vascular Anomalies‎ (ISSVA) [3, 6]. Now, it has recently been revised at the 20th ISSVA Workshop in Melbourne Australia, April 2014 [6]. So our case according to the recent literature study is termed as AVMs involving the lips.

Vascular tumors affecting the region of the head and neck are common, involving particularly the jaws. On the other hand, venous malformations (VMs) are rare but persistent and progressive in nature, and can be a fatal benign disease [1, 7]. Hemangiomas are the most common vascular tumors and should be separated from VMs because therapy is different for each patient [8]. Forbes et al. distinguished VMs as slow / low flow, and high / fast flow lesions based on angiographic presence of hemodynamic and contrast [8]. The first group comprises lymphatic malformations and VMs while the second group involves AVMs [8]. AVMs are the most violent type of VMs, which can lead to severe deformation and loss of functionality [9]. So we have specifically diagnosed using color doppler application.

The AVMs present a therapeutic challenge because of their hemodynamic characteristics and their growth modality [1]. They have to be treated according to their histopathology, location, and hemodynamic features as shown radiographically with angiography [8-9]. So we recommend to obtain angiography to find out the feeding vessel in this case before surgical interventions. AVMs may not need treatment when asymptomatic, but medication is needed if it is associated with discomfort, ulceration, bleeding, or heart problems. Multimodal treatment involving preoperative sclerosing agents or embolization accompanied by complete surgical resection remains the most traditional modern approach to treating such lesions [1-2]. Sometimes, surgical resection is associated with significant blood loss, and an incomplete resection also results in tumor regrowth to sizes that are much greater than its original size. The proximal ligation of the parent vessel is avoided because it is unsuccessful and can make the issue much more complex for potential endovascular therapy [1]. The patient was referred to a higher center for further investigation of angiography and management and all risk-related complications were described to the patient.

## Conclusions

The AVMs are fairly uncommon in the area of the head and neck. Because of their potential for uncontrollable bleeding, they may pose as a dental emergency while performing dental procedures such as tooth extraction, biopsy, or during the normal primary tooth exfoliation. They can cause troubling hemodynamic manifestations such as venous obstruction, distal ischemia, and high-performance cardiac failure. Therefore, these cases must be diagnosed early and treated promptly. A clear clinical background with imaging usually delineates the lesion well and provides an opportunity to make a cautious intervention decision. The surgical treatment is complicated and involves careful preparation and multidisciplinary approach.
